# Anorectal Pathophysiology in Solitary Rectal Ulcer Syndrome: Insights From a Cross-Sectional Study

**DOI:** 10.7759/cureus.97082

**Published:** 2025-11-17

**Authors:** Sudipta K Roy, Arka Banerjee, Bipul Barman

**Affiliations:** 1 Department of Gastroenterology, School of Digestive and Liver Diseases, Institute of Post Graduate Medical Education and Research (IPGMER) and Seth Sukhlal Karnani Memorial (SSKM) Hospital, Kolkata, IND

**Keywords:** anorectal manometry, balloon expulsion test, dyssynergic defecation, eastern india, functional bowel disorder, iapwg protocol, london classification, rectal hypersensitivity, rectoanal coordination, solitary rectal ulcer syndrome

## Abstract

Background and objectives

Solitary rectal ulcer syndrome (SRUS) is a rare, benign rectal disorder characterized by symptoms such as rectal bleeding, constipation, mucus discharge, and incomplete evacuation, often leading to diagnostic delays due to overlap with other colorectal conditions. Functional anorectal abnormalities are thought to contribute significantly to its pathogenesis, yet systematic evaluation remains limited. This study aimed to assess anorectal manometry (ARM) and balloon expulsion test (BET) findings in adult SRUS patients and to analyze their association with specific clinical symptoms.

Methods

This cross-sectional observational study included 24 adult patients with histologically confirmed SRUS from February 2023 to March 2024. Demographic and clinical data were recorded. All patients underwent ARM and BET. Findings were classified using London classification and normative Indian ARM parameters. Multiple correspondence analysis (MCA) and hierarchical clustering on principal components (HCPC) were used to identify symptom-pathophysiology patterns among SRUS patients. Univariable Firth's logistic regression explored associations between demographic variables, clinical features and ARM findings, while multivariable analysis was avoided due to small sample size.

Results

A total of 24 adult patients were included in the study: 12 (50%) males and 12 (50%) females. The median age was 26 years (range: 18-70). The most common symptoms were rectal bleeding (20; 83%), constipation (17; 71%), and mucus in stool (15; 63%). On ARM, the most frequent abnormalities were rectal hypersensitivity (10; 41.7%) and disorder of rectoanal coordination (9; 37.5%). Balloon expulsion was abnormal in nine (37.5%) patients. Multiple correspondence analysis identified two dimensions explaining 50.1% variance, distinguishing dysfunction severity (Dimension 1) and sensory-pressure phenotypes (Dimension 2). Hierarchical clustering revealed five distinct patient phenotypes: poor propulsion and dyssynergia phenotype (n=6, 25%), dyssynergia-dominant phenotype (n=4, 16.7%), hypersensitivity phenotype (n=7, 29.2%), normal phenotype (n=6, 25%), and isolated hypotension phenotype (n=1, 4.2%). Firth's logistic regression identified significant associations: constipation inversely predicted normal findings (OR=0.01, 95% CI: 0.00-0.10, p<0.001) and predicted rectal hypersensitivity (OR=21.00, 95% CI: 2.02-2885.43, p=0.007); digital rectal evacuation predicted abnormal expulsion with poor propulsion and dyssynergia (OR=25.00, 95% CI: 2.34-3457.52, p=0.005); disease duration predicted abnormal expulsion with dyssynergia (OR=1.08, 95% CI: 1.02-1.37, p=0.005).

Conclusion

SRUS patients show marked pathophysiological heterogeneity with distinct motor and sensory phenotypes. Constipation and digital evacuation strongly predict anorectal dysfunction. Objective ARM assessment is essential to identify these phenotypes and enable targeted therapies to optimize outcomes.

## Introduction

Solitary rectal ulcer syndrome (SRUS) is an uncommon, benign, chronic disorder of the rectum characterized by a spectrum of clinical, endoscopic, and histological findings. The reported incidence is estimated to be one per 100,000 persons annually, although underdiagnosis is common due to its heterogeneous presentation [1]. In one retrospective study of 80 patients, the median age at diagnosis was 48 years with a range of 14 to 76 years. Males and females appear to be affected equally, but a slight female preponderance has been suggested [2]. By contrast, in a series of 140 children, the majority (79%) were boys [3].

Clinically, SRUS manifests with per rectal bleeding, constipation, straining, excessive digital evacuation, mucus discharge, and abdominal pain, though the intensity and combination of symptoms vary [4]. The underlying pathophysiology is multifactorial, with rectal intussusception, rectal prolapse, paradoxical contraction of the puborectalis muscle, impaired rectal evacuation and chronic mucosal trauma playing central roles [5]. Repeated digital rectal evacuation, often used to relieve obstructed defecation, is recognized as a mechanical risk factor for solitary rectal ulcer syndrome, as it can cause direct mucosal trauma and perpetuate ulceration [6]. A hormonal influence has also been suggested, as illustrated by a case report of a woman with solitary rectal ulcer syndrome that completely resolved during two pregnancies but recurred in the non-pregnant state [7]. Defecatory disorders/evacuation disorders are increasingly recognized contributors [8,9], in line with the Rome IV criteria and the recent London Classification of anorectal disorders [10,11].

Endoscopic features of SRUS are variable, ranging from a single well-demarcated ulcer to multiple ulcers, erythema, or polypoid lesions [3]. Ulcers in SRUS are typically superficial, measuring 1-1.5 cm in diameter, though their size may vary from 0.5 cm to as large as 4 cm. Most lesions are located on the anterior rectal wall within 10 cm of the anal verge, although they may occasionally extend into the anal canal or even the sigmoid colon [12]. Histology remains the gold standard for diagnosis, typically showing fibromuscular obliteration of the lamina propria, hypertrophy of the muscularis mucosae, and glandular crypt abnormalities [5]. In polypoid variants, the mucosa often exhibits a villiform pattern, and in certain cases, glands may become displaced into the submucosa, a feature consistent with colitis cystica profunda [13].

Indian data on SRUS remain limited. Previous reports have documented typical symptoms and endoscopic features, with some studies also describing abnormalities on anorectal manometry (ARM), including high resting or squeeze anal pressures, rectal hypersensitivity, hyposensitivity, and dyssynergic defecation [9,14]. However, there is a paucity of data correlating these ARM findings with clinical features, and virtually no studies have addressed this issue from Eastern India, where access to manometry remains limited.

Given this gap, the present study aimed to evaluate the clinical, endoscopic, and manometric spectrum of SRUS patients in Eastern India, and to explore the association between ARM abnormalities and clinical symptoms. This could provide insight into the pathophysiological underpinnings of SRUS in this population and guide rational use of therapies such as biofeedback for evacuation disorders.

## Materials and methods

This was a cross-sectional observational study conducted at the Department of Gastroenterology, Institute of Post Graduate Medical Education and Research (IPGMER) and Seth Sukhlal Karnani Memorial (SSKM) Hospital, Kolkata, between February 2023 and March 2024. The study was approved by the Institutional Ethics Committee of IPGMER. Written informed consent was obtained from all participants prior to enrollment.

Adult patients (≥18 years) attending the gastroenterology outpatient department with endoscopically visible rectal lesions and histologically confirmed SRUS were included. SRUS was diagnosed on rectal biopsy showing fibromuscular obliteration of lamina propria, hypertrophy of muscularis mucosae with or without distortion of crypt architecture [1,5].

Patients were excluded if they had inflammatory bowel disease, prior pelvic surgery or obstetric injury, history of pelvic irradiation, spinal cord injury or neurocognitive disorders, psychiatric illness, pregnancy, or local anorectal sepsis (fistula/abscess).

A structured proforma was used to record demographic details, clinical features (per rectal bleeding, constipation, mucus discharge, digital evacuation, abdominal discomfort), and endoscopic findings.

Anorectal manometry and balloon expulsion test were performed using high-resolution water-perfused anorectal manometry (16-channel; Diversatek Healthcare, Milwaukee, WI, USA) following the International Anorectal Physiology Working Group (IAPWG) protocol. Findings were classified and reported using the London Classification of anorectal disorders [11]. Normative reference values for ARM parameters were derived from an Indian study on healthy volunteers by Deshmukh et al. [15].

All data were systematically recorded and entered into Microsoft Excel (Redmond, WA, USA). Categorical variables were expressed as numbers and percentages. Continuous variables were summarized as median with interquartile range (IQR) for non-normally distributed data. 

Multiple correspondence analysis (MCA) was performed to explore relationships among clinical features and anorectal manometry findings. All variables were coded as binary (Yes/No). MCA generates orthogonal dimensions that maximize variance explained in categorical data through optimal scaling.

Following MCA, hierarchical clustering on principal components (HCPC) was performed to identify discrete patient subgroups. Ward's hierarchical clustering method was applied to patient coordinates from the first two MCA dimensions. The optimal number of clusters was determined by examining the dendrogram structure, inertia gain at each agglomeration step, and clinical interpretability of resulting groups. A five-cluster solution was selected.

Cluster characteristics were described by calculating frequencies and percentages of clinical features and ARM findings within each cluster. Due to small sample size (n=24) and resulting small cluster sizes (n=1-7 per cluster), formal statistical comparisons between clusters were not performed. Clusters were visualized by projecting them onto the first two MCA dimensions.

Given the small sample size (n=24) and low event frequencies for ARM findings (ranging from 1 to 8 events per outcome), we performed univariable Firth's logistic regression to explore associations between demographic variables, clinical features, and ARM findings. Firth's penalized likelihood method was used to reduce small-sample bias and handle sparse data. Odds ratios with 95% profile likelihood confidence intervals and p-values are reported. P value <0.05 was considered statistically significant.

Multivariable modelling was not performed due to insufficient events per variable ratios (all outcomes had <10 events), which would result in unreliable estimates even with penalized methods.

All analyses were performed using R version 4.5.1 (R Foundation for Statistical Computing, Vienna, Austria) with packages FactoMineR, factoextra, logistf, and ggplot2.

## Results

A total of 24 adult patients with histologically confirmed SRUS were included in the study. The cohort comprised 12 (50%) males and 12 (50%) females. The median (IQR) age at presentation was 26 (20-39) years with a wide age range from 18 to 70 years. The median (IQR) of disease duration was 24 (12-57) months, ranging from six to 120 months. The median (IQR) interval from onset of symptoms to diagnosis was 20 (10-41) months, with a range of one to 72 months, reflecting a significant diagnostic delay in many patients (Tables 1, 2, 3).

**Table 1:** Baseline demographic characteristics N=24. Data are expressed as n (%) or median (IQR) as appropriate

Variables	Value
Gender (n (%))	
Male	12(50%)
Female	12(50%)
Total	24(100%)
Age in years	
Median (IQR)	26 (20-39)

**Table 2:** Disease duration

N	Median (in months)	IQR	Lowest value (in months)	Highest value (in months)
24	24	12-57	6	120

**Table 3:** Onset to diagnosis gap

N	Median (in months)	IQR	Lowest Value (in months)	Highest Value (in months)
24	20	10-41	1	72

The most frequent presenting symptom was rectal bleeding, reported by 20 (83%) patients. Constipation was present in 17 (71%) patients, mucus in stool in 15 (63%) patients, and digital rectal evacuation in 12 (50%) patients. Lower abdominal discomfort was the least common symptom, reported by four (17%) patients. On endoscopy, solitary ulcers were seen in 10 (41.6%) patients, whereas multiple ulcers were observed in 14 (58.3%) patients. No polypoid lesions or mucosal erythema were documented in this cohort (Tables 4, 5).

**Table 4:** Symptom distribution among patients with solitary rectal ulcer syndrome (SRUS)

Symptoms	n (%)
Rectal bleeding	20(83%)
Constipation	17(71%)
Mucus in stool	15(63%)
Digital rectal evacuation	12(50%)
Lower abdominal discomfort	4(17%)

**Table 5:** Endoscopic findings

Total number	Solitary ulcer (n (%))	Multiple ulcers (n (%))
24	10 (41.6%)	14 (58.3%)

On ARM, six (25%) patients had normal findings while the remaining 18 (75%) patients demonstrated abnormalities. Rectal hypersensitivity was the most frequent abnormality, observed in 10 (41.7%) patients. Disorder of rectoanal coordination was present in nine (37.5%) patients, rectal hyposensitivity in three (12.5%) patients, anal hypertension in three (12.5%) patients, and anal hypotension with normal contractility in one (4.2%) patient. Among those with disorder of rectoanal coordination, three (12.5%) patients showed abnormal expulsion with dyssynergia, while six (25%) patients had abnormal expulsion with poor propulsion and dyssynergia. Balloon expulsion testing was abnormal in nine (37.5%) of the study population (Tables 6, 7).

**Table 6:** Frequency of anorectal manometry (ARM) findings

Findings	n (%)
Normal	6 (25%)
Anal Hypotension with Normal Contractility	1 (4.2%)
Anal Hypertension	3 (12.5%)
Rectal Hyposensitivity	3 (12.5%)
Rectal Hypersensitivity	10 (41.7%)
Disorder of Rectoanal Coordination	9 (37.5%)

**Table 7:** Disorder of rectoanal coordination types

Findings	n (%)
Abnormal Expulsion with Dyssynergia	3 (12.5%)
Abnormal Expulsion with Poor Propulsion and Dyssynergia	6 (25%)

Distribution of all clinical symptoms and ARM findings is shown in Table 8. A significant proportion of the patients (17; 70.8%) experienced multiple concurrent symptoms. There were three patients (12.5%) with all five symptoms, eight patients (33.3%) with four symptoms, and two patients (8.3%) with three symptoms. A lower symptom burden was observed in the remaining patients, with four patients (16.7%) having two symptoms, and seven patients (29.2%) having a single symptom. The most frequent symptom complex, present in seven patients (29.2%), was the combination of rectal bleeding, constipation, mucus in stool, and the need for digital rectal evacuation.

**Table 8:** Anorectal manometry (ARM) findings in patients with various symptoms

ARM findings	Rectal bleed (n (%))	Constipation (n (%))	Mucus in stool (n (%))	Digital rectal evacuation (n (%))	Lower abdominal discomfort (n (%))
Normal	3(12.5%)	0(0%)	3(12.5%)	0(0%)	0(0%)
Anal Hypotension with Normal Contractility	0(0%)	0(0%)	1(4.2%)	0(0%)	0(0%)
Anal Hypertension	3(12.5%)	3(12.5%)	1(4.2%)	1 (4.2%)	0(0%)
Rectal Hyposensitivity	3(12.5%)	3(12.5%)	3(12.5%)	3(12.5%)	0(0%)
Rectal Hypersensitivity	10(41.7%)	10(41.7%)	5(20.8%)	6(25%)	2(8.3%)
Abnormal Expulsion with Dyssynergia	3(12.5%)	3(12.5%)	3(12.5%)	3(12.5%)	1(4.2%)
Abnormal Expulsion with Poor Propulsion and Dyssynergia	6(25%)	6(25%)	6 (25%)	6(25%)	2(8.3%)

Seven patients (29.2%) were found to have two or more distinct ARM findings. Six patients (25%) were found to have concurrent two ARM findings and one patient (4.2%) had concurrent three ARM abnormalities. The most frequent co-occurring ARM findings were the combination of abnormal expulsion with poor propulsion and dyssynergia along with rectal hypersensitivity, which were observed in two patients (8.3%).

**Figure 1:**
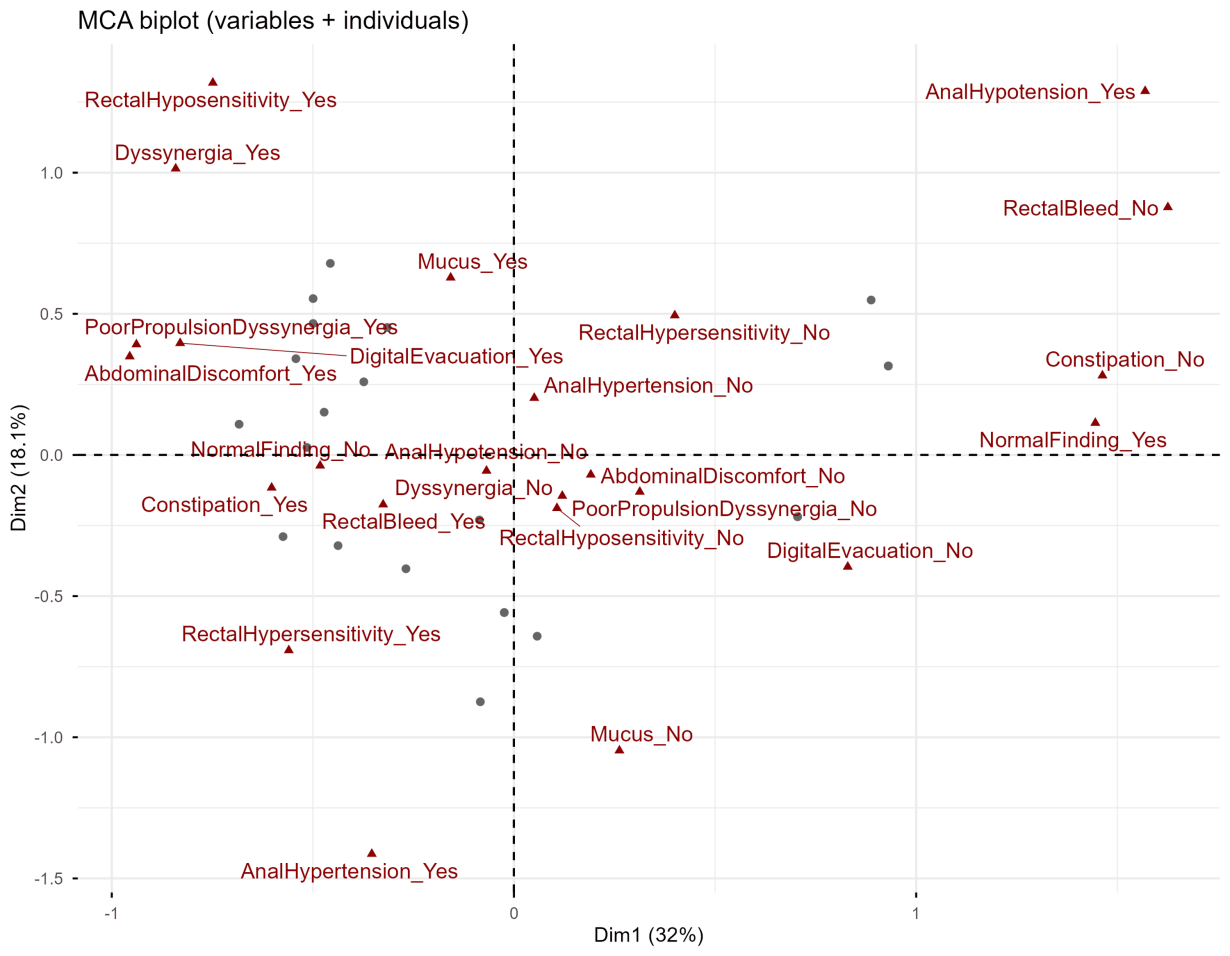
Multiple correspondence analysis (MCA) biplot of clinical and anorectal manometry (ARM) findings The biplot displays the results of the MCA, projecting both variable categories and individual patients onto a two-dimensional factor plane defined by the first two dimensions (Dim1 and Dim2). The percentage of inertia explained by each dimension is indicated on the axes. Each triangle represents a specific variable category (symptom or ARM finding), denoted by the suffix _Yes (present) or _No (absent). The position of a triangle indicates its contribution to the dimensions, and proximity between triangles indicates a strong association. Each dot represents an individual patient. The spatial proximity of a patient's dot to a specific variable triangle suggests that the patient is highly described by that characteristic. Abbreviations: Dim1=Dimension 1, Dim2=Dimension 2, RectalBleed=Rectal bleed, Mucus=Mucus in stool, DigitalEvacuation=Digital rectal evacuation, AbdominalDiscomfort=Lower abdominal discomfort, Dyssynergia=Abnormal expulsion with dyssynergia, PoorPropulsionDyssynergia=Abnormal expulsion with poor propulsion and dyssynergia, RectalHypersensitivity=Rectal hypersensitivity, RectalHyposensitivity=Rectal hyposensitivity, AnalHypertension=Anal hypertension, AnalHypotension=Anal hypotension with normal contractility, NormalFinding=Normal finding

We employed MCA to define clearer clinical subgroups of SRUS patients. MCA was performed using 12 categorical symptom and ARM variables. The first two dimensions accounted for 50.1% of total inertia (Dimension 1: 32.0%, Dimension 2: 18.1%). MCA biplot (Figure 1) demonstrated the negative side of Dimension 1 was characterized by presence of constipation, digital rectal evacuation, lower abdominal discomfort, rectal bleeding, abnormal expulsion with poor propulsion and dyssynergia. In contrast, the positive side was characterized by absence of these symptoms, along with normal ARM findings. The positive Dimension 2 was associated with abnormal expulsion with dyssynergia and rectal hyposensitivity, while the negative Dimension 2 was associated with anal hypertension and rectal hypersensitivity.

**Figure 2:**
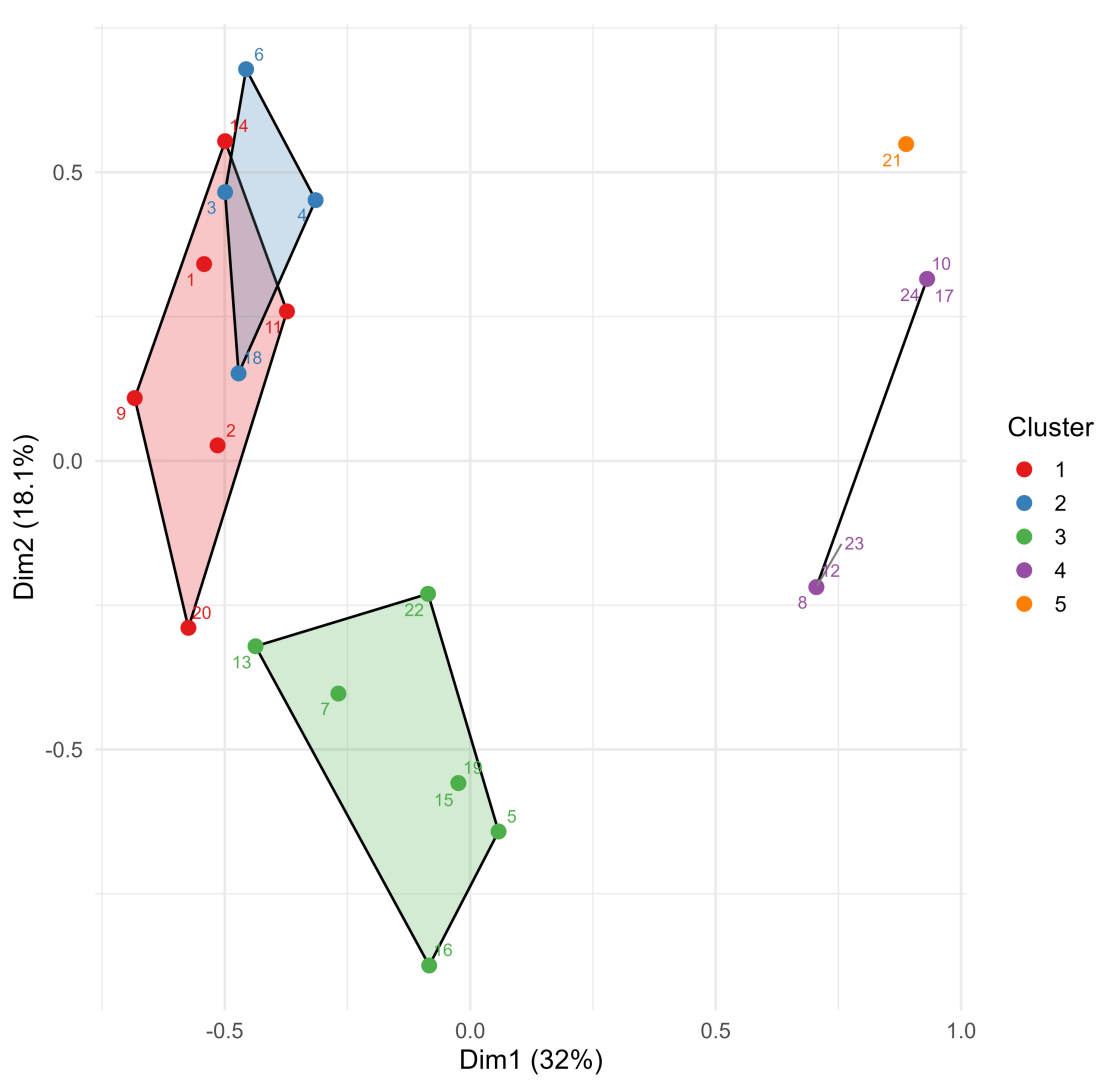
Hierarchical clustering on principal components (HCPC) showing solitary rectal ulcer syndrome (SRUS) patient clusters The figure shows individual patients projected on the first two multiple correspondence analysis (MCA) dimensions, with convex polygons enclosing cluster boundaries. The numbers represent individual patients.

HCPC was applied to the first two MCA dimensions in order to identify discrete patient subgroups. Five distinct clusters emerged (Figure 2), characterized by specific symptom-pathophysiology profiles (Table 9). Cluster 1 (n=6, 25%): All patients (100% of Cluster 1) presented with rectal bleeding, constipation, mucus in stool, digital rectal evacuation, and abnormal expulsion with poor propulsion and dyssynergia. Half of the patients (50% of Cluster 1) had rectal hypersensitivity. Cluster 2 (n=4, 16.7%): All patients (100% of Cluster 2) had rectal bleeding, constipation, mucus, and digital rectal evacuation. Three-quarters of the patients (75% of Cluster 2) exhibited abnormal expulsion with dyssynergia and half of the patients (50% of Cluster 2) showed rectal hyposensitivity. Cluster 3 (n=7, 29.2%): All patients (100% of Cluster 3) reported rectal bleeding and constipation, but mucus in stool (14.3% of Cluster 3) and digital rectal evacuation (28.6% of Cluster 3) were less common. 85.7% of the Cluster 3 had rectal hypersensitivity and 28.6% had anal hypertension. Cluster 4 (n=6, 25%): All patients (100% of Cluster 4) had normal ARM findings with half of the patients (50% of Cluster 4) reporting rectal bleeding and mucus. Cluster 5 (n=1, 4.2%): A single patient with anal hypotension with normal contractility as the sole ARM abnormality, with mucus as the only symptom.

**Table 9:** Cluster-wise distribution of symptoms and anorectal manometry (ARM) findings Percentages of each cluster row are expressed using the total number of the patients in that cluster as the denominator (100%)

Cluster	n	Rectal bleed (n(%))	Constipation (n(%))	Mucus in stool (n(%))	Digital rectal evacuation (n(%))	Lower abdominal discomfort (n(%))	Abnormal expulsion with dyssynergia (n(%))	Abnormal expulsion with poor propulsion and dyssynergia (n(%))	Rectal hypersensitivity (n(%))	Rectal hyposensitivity (n(%))	Anal hypertension (n(%))	Anal hypotension with normal contractility (n(%))	Normal finding (n(%))
1	6	6 (100%)	6 (100%)	6 (100%)	6 (100%)	2 (33.3%)	0 (0%)	6 (100%)	3 (50%)	1 (16.7%)	1 (16.7%)	0 (0%)	0 (0%)
2	4	4 (100%)	4 (100%)	4 (100%)	4 (100%)	1 (25%)	3 (75%)	0 (0%)	1 (25%)	2 (50%)	0 (0%)	0 (0%)	0 (0%)
3	7	7 (100%)	7 (100%)	1 (14.3%)	2 (28.6%)	1 (14.3%)	0 (0%)	0 (0%)	6 (85.7%)	0 (0%)	2 (28.6%)	0 (0%)	0 (0%)
4	6	3 (50%)	0 (0%)	3 (50%)	0 (0%)	0 (0%)	0 (0%)	0 (0%)	0 (0%)	0 (0%)	0 (0%)	0 (0%)	6 (100%)
5	1	0 (0%)	0 (0%)	1 (100%)	0 (0%)	0 (0%)	0 (0%)	0 (0%)	0 (0%)	0 (0%)	0 (0%)	1 (100%)	0 (0%)

We analyzed 56 associations between demographic variables, symptoms and ARM findings using Firth's penalized likelihood univariable logistic regression in our cohort of 24 patients (Appendix). Seven associations demonstrated statistical significance (p < 0.05), with effects observed for both protective and risk factors (Table 10).

**Table 10:** Significant associations between demographic variables, symptoms and anorectal manometry (ARM) findings using Firth's penalized univariable logistic regression OR = Odds Ratio; CI = Confidence Interval. Significant associations:  ***p < 0.001, **p < 0.01, *p < 0.05.

ARM Findings	Demographic variables or symptoms	OR (95% CI)	P-value
Abnormal Expulsion with Dyssynergia	Disease duration	1.08 (1.02-1.37)	0.005**
Normal findings	Constipation	0.01 (0.00-0.10)	<0.001***
Normal findings	Digital rectal evacuation	0.04 (0.00-0.43)	0.005**
Normal findings	Rectal bleeding	0.09 (0.01-0.70)	0.022*
Abnormal Expulsion with Poor Propulsion and Dyssynergia	Digital rectal evacuation	25.00 (2.34-3457.52)	0.005**
Abnormal Expulsion with Poor Propulsion and Dyssynergia	Mucus in stool	13.00 (1.23-1786.25)	0.030*
Rectal Hypersensitivity	Constipation	21.00 (2.02-2885.43)	0.007**

Constipation showed a strong inverse association with normal manometry findings (OR = 0.01, 95% CI: 0.00-0.10, p < 0.001), while being positively associated with rectal hypersensitivity (OR = 21.00, 95% CI: 2.02-2885.43, p = 0.007). Digital rectal evacuation demonstrated a strong positive association with abnormal expulsion with poor propulsion and dyssynergia (OR = 25.00, 95% CI: 2.34-3457.52, p = 0.005) but a protective association against normal findings (OR = 0.04, 95% CI: 0.00-0.43, p = 0.005). Longer disease duration was associated with increased odds of abnormal expulsion with dyssynergia (OR = 1.08, 95% CI: 1.02-1.37, p = 0.005). Mucus in stool was associated with abnormal expulsion with poor propulsion and dyssynergia (OR = 13.00, 95% CI: 1.23-1786.25, p = 0.030), and rectal bleeding showed a protective effect against normal findings (OR = 0.09, 95% CI: 0.01-0.70, p = 0.022).

## Discussion

In this cross-sectional study, 24 adult patients with SRUS were systematically evaluated with clinical, endoscopic, and anorectal physiological assessments. The median age of presentation was 26 years, younger than the mean age of 41 ± 19 years reported by Behera et al. [14], and lower than the median age of 48 years described by Tjandra et al. [2]. Gender distribution was equal in our cohort, whereas previous studies have reported a slight female preponderance (female: male ratio 1.4:1 in the series by Tjandra et al. [2]) and a male predominance in the north Indian study by Behera et al. [14].

The disease course was prolonged in our patients, with a median duration of 24 months and a median delay in diagnosis of 20 months. This is consistent with the observations by Martin et al. [1], who reported an average delay of 42 months between symptom onset and diagnosis. Such delays highlight the non-specific nature of clinical features and the frequent overlap of SRUS with other colorectal disorders.

Symptomatically, rectal bleeding was the most frequent complaint in our study (20; 83%), comparable to Behera et al., who reported rectal bleeding in 83.7% of cases [14], and lower as compared to the study done by Martin et al., which showed a rectal bleeding rate of 98% [1]. Constipation was observed in 17 (71%) of our patients, higher than the 46.7% reported by Behera et al. [14] and 49% in the series by Martin et al. [1]. Mucus discharge was present in 15 (63%) of our cohort, compared with 28.8% and 96% described by Behera et al. [14] and Martin et al. [1], respectively.

Endoscopic evaluation in our patients revealed solitary ulcers in 10 (42%) and multiple ulcers in 14 (58%). Martin et al. [1] reported solitary ulcers in 57% of cases, with additional findings such as polypoid lesions in 25% and hyperemic mucosa in 18%. Behera et al. [14] similarly found solitary and multiple ulcers in 44.6% each, with polypoid lesions in 17%. The absence of polypoid or hyperemic lesions in our study suggests a more ulcerative disease phenotype in this population.

Functional assessment with anorectal manometry demonstrated rectal hypersensitivity in 10 (41.7%) and disorder of rectoanal coordination in nine (37.5%) of patients. Sharma et al. [9] reported functional defecation disorder in 43% of SRUS patients, while Jain et al. [16] observed dyssynergic defecation in 75%. The prevalence of abnormal balloon expulsion was nine (37.5%) in our study, slightly lower as compared to 53% reported in both Sharma et al. [9] and Behera et al. [14].

The MCA identified distinct patient subgroups based on symptom-pathophysiology profiles. Dimension 1 represents severity of dysfunction, with implications for biofeedback therapy. Dimension 2 shows separation of sensory/pressure phenotypes, which suggests different underlying neuromuscular mechanisms that may require tailored therapeutic approaches. HCPC on principal components of MCA identified five patient clusters. The clusters demonstrate substantial heterogeneity in pathophysiological mechanisms underlying similar presenting symptoms. Cluster 1 (poor propulsion and dyssynergia phenotype, n=6) represents the most severe motor dysfunction, with all patients exhibiting both rectal propulsive failure and sphincter dyssynergy. These patients universally required digital rectal evacuation (100% of Cluster 1), consistent with mechanical inability to evacuate. Treatment for this group likely requires multimodal approaches addressing both rectal contractility (prokinetic agents, rectal stimulation protocols) and pelvic floor coordination (biofeedback therapy). Cluster 2 (dyssynergia dominant phenotype, n=4) showed isolated sphincter incoordination (75% of Cluster 2) without major propulsive deficits, suggesting preserved rectal function with impaired sphincter relaxation. These patients may be optimal candidates for standard pelvic floor biofeedback protocols, as their primary dysfunction is behavioral sphincter incoordination rather than propulsive failure. Cluster 3 (hypersensitivity phenotype, n=7, the largest group) exhibited rectal hypersensitivity (85.7% of Cluster 3) without any motor dysfunction, despite universal constipation (100% of Cluster 3). This hypersensitivity-constipation paradox is likely due to enhanced perception of rectal distention leading to incomplete evacuation attempts. These patients may benefit from neuromodulatory interventions or medications targeting visceral hypersensitivity rather than traditional prokinetic or behavioral therapies. Cluster 4 (normal phenotype, n=6) had structurally normal anorectal function (100% of Cluster 4) despite residual symptoms of bleeding (50% of Cluster 4) and mucus (50% of Cluster 4) without constipation. This suggests mucosal inflammation or irritation as the symptom driver rather than motor or sensory dysfunction. These patients may require colonoscopic evaluation and management of underlying mucosal pathology rather than functional treatments. Cluster 5 (isolated hypotension phenotype, n=1) represents either a rare phenotype or sampling variability. Anal hypotension with minimal symptoms is unusual and warrants detailed evaluation for sphincter injury or neurological conditions in larger cohorts. The clear separation of poor propulsion dyssynergia (Cluster 1) from isolated dyssynergia (Cluster 2) is particularly clinically relevant, as these have previously been considered variants of the same disorder. Our findings suggest they may represent distinct pathophysiological entities requiring different therapeutic approaches.

To complement the multivariate phenotyping, univariable Firth's penalized logistic regression was performed to assess specific predictor-outcome relationships. This analysis identified several significant, clinically meaningful associations between demographic variables, symptoms and specific anorectal manometry patterns. Disease duration emerged as a significant predictor of abnormal expulsion with dyssynergia (OR=1.08, 95% CI: 1.02-1.37, p=0.005), suggesting progressive pelvic floor dysfunction with chronicity or development of maladaptive compensatory mechanisms. This temporal relationship was not apparent in the cross-sectional MCA but emerged through quantitative regression analysis. This temporal relationship merits longitudinal investigation to determine whether early intervention might alter disease course. Constipation showed the strongest association with abnormal ARM findings. The inverse association between constipation and normal findings was strong (OR=0.01, 95% CI: 0.00-0.10, p<0.001), indicating that constipated patients had 99% reduced odds of normal anorectal physiology. Conversely, constipation powerfully predicted rectal hypersensitivity (OR=21.00, 95% CI: 2.02-2885.43, p=0.007). This bidirectional finding validates the MCA result showing constipation as a hub variable connecting to multiple ARM abnormalities and supports the hypersensitivity-constipation paradox (Cluster 3). The wide confidence intervals reflect a small sample size, but the direction and magnitude of effects are clinically meaningful. Digital rectal evacuation emerged as a behavioral marker of severe dysfunction. Patients requiring digital rectal evacuation had 25-fold increased odds of abnormal expulsion with poor propulsion and dyssynergia (OR=25.00, 95% CI: 2.34-3457.52, p=0.005) but 96% reduced odds of normal findings (OR=0.04, 95% CI: 0.00-0.43, p=0.005). This validates the MCA result and explains why Clusters 1 and 2 (which universally exhibited digital rectal evacuation) demonstrated severe motor dysfunction. Digital rectal evacuation thus serves as a clinical indicator for triaging patients toward anorectal manometry assessment. Rectal bleeding inversely predicted normal findings (OR=0.09, 95% CI: 0.01-0.70, p=0.022), indicating that bleeding patients rarely had entirely normal physiology. However, bleeding did not specifically predict any particular dysfunction pattern, consistent with its distribution across multiple clusters in HCPC. This suggests bleeding may be a non-specific marker of anorectal disorder rather than a phenotype-specific symptom. Mucus in stool predicted poor propulsion (OR=13.00, 95% CI: 1.23-1786.25, p=0.030), suggesting that mucus may reflect chronic rectal distention or incomplete evacuation rather than purely mucosal pathology. This association was less apparent in MCA but emerged in focused regression analysis.

The convergence of univariable regression, MCA, and HCPC results strengthens confidence in key associations. Three findings emerged consistently across analytical approaches. First, constipation emerged as a cardinal symptom - appearing as a central variable in MCA, cluster analyses, and as the strong regression predictor (OR = 21 for hypersensitivity; OR = 0.01 for normal findings). This highlights its value as the principal indicator for anorectal physiological assessment. Second, digital rectal evacuation identified severe motor dysfunction across MCA positioning (negative Dim1), cluster characteristics (100% in Clusters 1-2), and regression (OR=25 for poor propulsion dyssynergia). Clinically, this suggests that eliciting digital rectal evacuation history should prompt targeted evaluation for dyssynergia and propulsive disorders. Third, the separation of sensory/pressure phenotypes along MCA Dimension 2 and cluster differentiation (Cluster 3's hypersensitivity without dyssynergia) were quantitatively confirmed by constipation predicting hypersensitivity (OR=21) independent of motor findings. This validates the existence of a distinct sensory phenotype requiring different therapeutic approaches than motor dysfunction.

These findings have practical implications for clinical assessment. A brief two-question screening based on the presence of constipation and the need for digital rectal evacuation may effectively identify patients who require ARM evaluation. Furthermore, the phenotypic heterogeneity identified argues against uniform treatment protocols. Cluster 1 patients (poor propulsion, requiring prokinetics, sacral nerve stimulation, biofeedback) differ fundamentally from Cluster 3 patients (hypersensitivity, requiring neuromodulation) despite both presenting with constipation. Current treatment algorithms based on symptoms alone may fail to match therapy to underlying physiology, potentially explaining variable treatment responses in clinical practice.

Strengths of the study include multi-method analytical convergence (MCA, HCPC, Firth's regression), statistically rigorous approaches for sparse data (penalized likelihood), and symptom-physiology integration, which revealed clinically meaningful clusters of biologically plausible phenotypes with clear therapeutic implications. The study provides preliminary data essential for larger investigations.

Critical limitations include small sample size severely constraining power and precision; very small clusters (n=1-7) with unassessed stability; extremely wide confidence intervals in Firth regression; inability to perform multivariable modeling preventing confounder adjustment; multiple testing without correction increasing false positive risk; single-center referral population limiting generalizability; cross-sectional design, absence of a control group, precluding causality; absence of follow up treatment response data; and the use of laboratory balloon expulsion testing instead of commode-based testing. All findings should be interpreted as exploratory and hypothesis-generating, requiring validation in larger cohorts before clinical application.

## Conclusions

SRUS patients exhibit substantial pathophysiological heterogeneity with five distinct phenotypes. Constipation and digital rectal evacuation are powerful predictors of anorectal dysfunction. Multivariate analysis demonstrates that identical symptoms arise from different underlying mechanisms-emphasizing the need for objective ARM assessment. Early identification of specific phenotypes may guide targeted therapies such as biofeedback for coordination disorders or neuromodulation for hypersensitivity, potentially improving treatment outcomes.

## Appendices

**Table 11:** Associations between all demographic variables, symptoms and anorectal manometry (ARM) findings using Firth's penalized univariable logistic regression OR = Odds Ratio; CI = Confidence Interval. Significant associations: ***p < 0.001, **p < 0.01, *p < 0.05.

Outcome	Predictor	OR (95% CI)	P-value	Significance
Anal hypertension	Age	0.95 (0.71–1.05)	0.398	
Anal hypertension	Constipation	3.62 (0.29–511.58)	0.356	
Anal hypertension	Digital rectal evacuation	0.55 (0.04–4.82)	0.585	
Anal hypertension	Disease duration	1.00 (0.94–1.03)	0.954	
Anal hypertension	Female sex	0.55 (0.04–4.82)	0.585	
Anal hypertension	Lower abdominal discomfort	0.56 (0.00–7.53)	0.698	
Anal hypertension	Mucus in stool	0.31 (0.02–2.76)	0.289	
Anal hypertension	Rectal bleeding	1.80 (0.13–258.90)	0.698	
Anal hypotension	Age	1.07 (0.97–1.20)	0.159	
Anal hypotension	Constipation	0.12 (0.00–2.62)	0.178	
Anal hypotension	Digital rectal evacuation	0.31 (0.00–6.36)	0.452	
Anal hypotension	Disease duration	1.02 (0.97–1.07)	0.271	
Anal hypotension	Female sex	0.31 (0.00–6.36)	0.452	
Anal hypotension	Lower abdominal discomfort	1.44 (0.01–32.33)	0.835	
Anal hypotension	Mucus in stool	1.97 (0.09–299.38)	0.675	
Anal hypotension	Rectal bleeding	0.06 (0.00–1.28)	0.071	
Abnormal expulsion with Dyssynergia	Age	1.07 (1.00–1.17)	0.061	
Abnormal expulsion with Dyssynergia	Constipation	3.62 (0.29–511.58)	0.356	
Abnormal expulsion with Dyssynergia	Digital rectal evacuation	9.21 (0.76–1296.84)	0.086	
Abnormal expulsion with Dyssynergia	Disease duration	1.08 (1.02–1.37)	0.005	**
Abnormal expulsion with Dyssynergia	Female sex	1.82 (0.21–22.43)	0.585	
Abnormal expulsion with Dyssynergia	Lower abdominal discomfort	3.17 (0.24–33.17)	0.347	
Abnormal expulsion with Dyssynergia	Mucus in stool	5.32 (0.43–748.77)	0.214	
Abnormal expulsion with Dyssynergia	Rectal bleeding	1.80 (0.13–258.90)	0.698	
Normal findings	Age	0.99 (0.91–1.05)	0.796	
Normal findings	Constipation	0.01 (0.00–0.10)	<0.001	***
Normal findings	Digital rectal evacuation	0.04 (0.00–0.43)	0.005	**
Normal findings	Disease duration	0.98 (0.92–1.02)	0.334	
Normal findings	Female sex	2.22 (0.38–15.54)	0.374	
Normal findings	Lower abdominal discomfort	0.25 (0.00–2.91)	0.305	
Normal findings	Mucus in stool	0.52 (0.08–3.12)	0.466	
Normal findings	Rectal bleeding	0.09 (0.01–0.70)	0.022	*
Abnormal expulsion with Poor propulsion and dyssynergia	Age	0.98 (0.89–1.05)	0.597	
Abnormal expulsion with Poor propulsion and dyssynergia	Constipation	8.48 (0.79–1167.29)	0.083	
Abnormal expulsion with Poor propulsion and dyssynergia	Digital rectal evacuation	25.00 (2.34–3457.52)	0.005	**
Abnormal expulsion with Poor propulsion and dyssynergia	Disease duration	0.95 (0.86–1.00)	0.064	
Abnormal expulsion with Poor propulsion and dyssynergia	Female sex	1.00 (0.17–5.93)	1.000	
Abnormal expulsion with Poor propulsion and dyssynergia	Lower abdominal discomfort	3.67 (0.45–31.24)	0.215	
Abnormal expulsion with Poor propulsion and dyssynergia	Mucus in stool	13.00 (1.23–1786.25)	0.030	*
Abnormal expulsion with Poor propulsion and dyssynergia	Rectal bleeding	4.03 (0.34–564.56)	0.305	
Rectal hypersensitivity	Age	1.00 (0.95–1.06)	0.858	
Rectal hypersensitivity	Constipation	21.00 (2.02–2885.43)	0.007	**
Rectal hypersensitivity	Digital rectal evacuation	1.89 (0.40–9.70)	0.426	
Rectal hypersensitivity	Disease duration	1.00 (0.97–1.03)	0.954	
Rectal hypersensitivity	Female sex	0.53 (0.10–2.53)	0.426	
Rectal hypersensitivity	Lower abdominal discomfort	1.47 (0.19–11.40)	0.700	
Rectal hypersensitivity	Mucus in stool	0.43 (0.08–2.13)	0.301	
Rectal hypersensitivity	Rectal bleeding	9.00 (0.80–1250.30)	0.080	
Rectal hyposensitivity	Age	1.04 (0.97–1.12)	0.241	
Rectal hyposensitivity	Constipation	3.62 (0.29–511.58)	0.356	
Rectal hyposensitivity	Digital rectal evacuation	9.21 (0.76–1296.84)	0.086	
Rectal hyposensitivity	Disease duration	1.03 (0.99–1.07)	0.141	
Rectal hyposensitivity	Female sex	1.82 (0.21–22.43)	0.585	
Rectal hyposensitivity	Lower abdominal discomfort	0.56 (0.00–7.53)	0.698	
Rectal hyposensitivity	Mucus in stool	5.32 (0.43–748.77)	0.214	
Rectal hyposensitivity	Rectal bleeding	1.80 (0.13–258.90)	0.698	
